# The Observer

**Published:** 1881-05

**Authors:** 


### THE OBSERVER: INTRODUCTORY.

*The Observer* opposes the International Hahnemannian Association "with all its strength and heart." First, and chiefly, because their pharmacology is not the pharmacology of Hahnemann, and because the preparations upon which Hahnemann relied (from the tincture to the 30th centesimal) were ignored by the International Association. Here is the great stumbling-block in the way of *The Observer*, and no doubt the chief objection to the existence of the International Hahnemannian Association. *The Observer* does not approve of high dilutions. It requires too much care and study to apply the exact medicine to the disease it would cure. That close individualization is difficult, and therefore distasteful, to *The Observer*. *The Observer* does not like the higher mathematics of our profession, and therefore takes its stand upon what it assumes to be the practice of Hahnemann.

The International Association believe the principle of medicine declared by Hahnemann will stand while the world standeth; therefore they declared that they "believe the 'Organon of the Healing Art' as promulgated by Samuel Hahnemann, the only reliable guide in therapeutics." Though principles could not alter, the application of the principle may be altered and improved by experience.

It is said, in the hands of a mummy entombed in a catacomb for a thousand years there have been found grains, which being planted have brought forth fruit; and we believe that in the dead hand of every great leader of his age—a pivotal man—will be found a living principle which the generations that follow may plant, cultivate and produce greater fruit. That is the principle of the progress of the human race.

But *The Observer* would close the catacomb, sit down at the tomb, and ignore the offering of the good held in hand for its acceptance.

*The Observer* has not followed Hahnemann in his late teachings, but is willing to halt at his beginnings.

Hahnemann, in his "Medicine of Experience" says, "None but the careful observer can have any idea of the height to which the sensitiveness of the body to medicinal irritation is increased in a state of disease. It exceeds all belief, when the disease has attained a great intensity. An insensible, prostrated, comatose typhus patient, unroused by any shaking, deaf to all calling, will be rapidly restored to consciousness by the smallest dose of opium, were it a million times smaller than any mortal ever yet prescribed." From this it may be clearly seen that Hahnemann put no limit to the extent of dilution.

The second objection made by *The Observer* against the International Hahnemannian Association is in these words. "Because some of them would introduce into Materia Medica many vile and repulsive substances falsely called medicines; preparations never having the sanction of Hahnemann; such things as Syphilinin, Gonorrhœin, Leucorrhœain, Carcinomatin, Hydrophobin, Dysenterin, etc., etc., the discharge of vilest ulcers, and the most fœtid excrements. These may belong to isopathy, but do not to homœopathy, and the endorsement of the most pharisaic purist can not make them legitimate."

*The Observer* speaks of the substances introduced, as it asserts, by the International Hahnemannian Association, with such intense disgust, such squeamish, over nice sensibility as they pass in review before its imagination, as to cause us as much surprise as if we had heard of a man-of-war's man taking fright at a mouse.

It says these things belong to isopathy not to homœopathy. But we would ask *The Observer* if these substances should produce in the healthy body a series of specific, morbid symptoms, and recorded whether their application to similar disease would not be strictly homœopathic?

But we utterly deny that the International Hahnemannian Association have ever in the statement of principles at the formation of the society, or in the principles themselves, ever proposed or alluded to the introduction of the substances above named into the Materia Medica or practice.

*The Observer* says such preparations have never had the sanction of Hahnemann. Here *The Observer* is greatly mistaken. In the "Medicine of Experience," Hahnemann says, "Every simple medicinal substance like the specific, morbific miasmata (small-pox, measles, the venom of vipers, the saliva of rabid animals, etc)., cause a peculiar specific disease, a series of determinate symptoms which is not produced precisely in the same way by another medicine in the world. In this way we must obtain a knowledge of a sufficient supply of artificial agents (medicines) for curative implements, so that we may be able to make a selection from among them."

The nervous condition of *The Observer* in considering these substances brings to mind very forcibly a scene in Shakspeare's "Henry the Fourth," between Hotspur and the king, and that we may hold a mirror before *The Observer*, that it may see itself, we quote it.

> "But, I remember, when the fight was done,
> When I was dry with rage and extreme toil,
> Breathless and faint, leaning upon my sword,
> Came there a certain lord, neat, trimly dress'd,
> Fresh as a bridegroom; and his chin, new reap'd
> Show'd like a stubble-land at harvest home;
> He was perfumed like a milliner;
> And 'twixt his finger and his thumb he held
> A pouncet-box, which ever and anon
> He gave his nose and took't away again;—
> Who, therewith angry, when it next came there,
> Took it in snuff:—and still he smil'd and talk'd;
> And, as the soldiers bore dead bodies by,
> He call'd them—untaught knaves, unmannerly

To bring a slovenly unhandsome corse
Betwixt the wind and his nobility.
With many holiday and lady terms
He question'd me; * * * * * *
I then, all smarting, with my wounds being cold,
To be so pester'd with a popinjay,
Answer'd neglectingly, * * * * *
* * * * * * * for he made me mad,
To see him shine so brisk, and smell so sweet,
And talk so like a waiting-gentlewoman,
Of guns, and drums, and wounds, (God save the mark)!
And telling me, the sovereign'st thing on earth
Was parmaceti, for an inward bruise;
And that it was great pity, so it was,
That villainous salt-petre should be digg'd
Out of the bowels of the harmless earth,
Which many a good tall fellow had destroy'd
So cowardly; and, but for these vile guns,
He would himself have been a soldier."

"INTERNATIONAL."

